# Mental health and workplace factors: comparison of the Ghanaian and Australian mining industry

**DOI:** 10.1186/s12913-022-07712-0

**Published:** 2022-03-10

**Authors:** Asare-Doku Winifred, Rich Louise Jane, Kelly Brian, Kwesi Amponsah-Tawiah, James Carole

**Affiliations:** 1 School of Medicine and Public Health, College of Health, Medicine and Wellbeing, The University of Newcastle, Callaghan, NSW 2308 Australia; 2 Centre for Resources Health and Safety, The University of Newcastle, Callaghan, NSW 2308 Australia; 3 Centre for Brain and Mental Health Research, The University of Newcastle, Callaghan, NSW 2308 Australia; 4 National Drug and Alcohol Research Centre, University of New South Wales, Randwick, NSW Australia; 5 Department of Organisation & Human Resource Management, University of Ghana Business School, Legon-Accra, Ghana; 6 School of Health Sciences, College of Health, Medicine and Wellbeing, The University of Newcastle, Callaghan, NSW 2308 Australia

**Keywords:** Mining, Mental health, Psychological distress, Workplace, Alcohol, Job demand, Job control

## Abstract

**Background:**

Mining is a global industry and contributes significantly to international economies. This study seeks to compare the patterns of psychological distress, job demand-control, and associated characteristics between two countries (Australia/Ghana) to increase understanding of cross-cultural factors relevant to mental health in this industry.

**Method:**

A cross-sectional study design was used. Eight coal mines in Australia and five gold mines in Ghana. A total of 2622 mineworkers participated in this study. Kessler Psychological Distress Scale (K10), Job Content Questionnaire (JCQ), Alcohol Use Disorders Identification Test (AUDIT), the Berkman-Syme Social Network Index (SNI) and help-seeking questionnaire.

**Results:**

Ghanaian mineworkers reported increased psychological distress compared to Australian mineworkers; Job demands outweighed control among Ghanaian mineworkers but was associated with lower risk of psychological distress compared to Australian mineworkers; Ghanaian mineworkers were significantly less likely to drink alcohol at risky levels but this was associated with higher psychological distress; Increased social network was associated with decreased psychological distress for both countries.

**Conclusions:**

These findings identify cultural and geographical differences in the socio-demographics, workplace factors, psychological distress, and alcohol use in both countries. Cross-cultural occupational workplace factors and mental health issues are highlighted. Potential workplace interventions applicable in comparable settings are recommended.

## Introduction

There are similarities between mining in Ghana and Australia. Mining is a large employer in both countries contributing significantly to their respective economies. Mining companies in Ghana directly employ on average 7000 people annually, while in total approximately 111,000 jobs are supported [1]. In Australia, mining employs 242,800 workers accounting for 1.9% of the total workforce [2]. Gold mining is the largest industry in Ghana and coal mining the largest in Australia. Gold is the leading mineral in revenue generation in Ghana accounting for 93.28% of gross mineral revenue [3]. Australia is one of the world’s largest producers and exporters of coal. Coal was the highest earning export commodity in 2019 accounting for $66 billion in export revenue in Australia [2]. In 2018, the value of coal exports was $67 billion, equivalent to 3½ per cent of nominal GDP [4].

There are also significant differences between the two countries. In Australia, FIFO (Fly-In-Fly-Out) and DIDO (Drive-In-Drive-Out) are features of many mining operations in remote locations [5]. Anecdotal evidence suggests most of the unskilled labour force in Ghana are from the local mining communities with a minority DIDO skilled labour force from the cities [6]. In addition, Australia has active mental health policies and structures in the workplace; mental health policies are incorporated in the health and safety policies in the Australian mining industry [7]. Ghana is in the early stages of developing mental health policies in the workplace. Australia is a high-income country and Ghana is a lower-middle income country hence specific contextual factors are present in both countries [8].

Mental health is an essential part of creating a safe and healthy workplace. Although work is beneficial for the mental health of adults, there is evidence that certain features of the workplace can affect workers’ mental health adversely and increase the likelihood of the occurrence of a mental illness [9]. Psychosocial hazards such as increased work demands and low levels of job control potentially affect the mental health of workers [10]. The effect of these are likely to lead to errors in judgement, sleep problems, depression, anxiety, excessive drinking, distraction during work and workplace injury [11]. The workplace can assist in the identification of mental health problems, and the facilitation of mental health interventions.

The World Health Organization recommends that international guidelines for a healthy workplace be tailored to specific sectors and cultures [12]. It is therefore important to develop governmental legislation, strategies, and workplace policies to guide this [12]. Ghana has a Mental Health Act (Act 846 of 2012) which is yet to be implemented, the government is working towards the establishment of a mental health fund to help with implementation of the policy. There is no legislation or policy regarding workplace mental health. Countries such as Australia, the USA, UK, and the European Union (EU) have developed policies and legislations towards a mentally healthy workplace [13]. In Australia, employers are obliged to take appropriate steps in identifying workplace features that contribute to mental illness of workers and initiate actions to minimise these risks [14].

Australia has demonstrated a global leadership role in mental health in the mining industry. A guideline for mental health and well-being has been developed to support the Australian mining industry response to workplace mental health [15]. With no such active policies and framework regarding mental health in Ghana’s resource sector, thus, opportunities exist to learn from countries that have acted in this regard and tailor strategies to the Ghanaian context [15]. This study examines the patterns of psychological distress, job demand-control, and associated characteristics among mineworkers in Ghana and Australia. The study will enhance understanding of the role of culture in the experience and handling of mental health issues in both countries.

## Methods

### Study design and population

The design for the study was cross-cultural comparative research design [16]. Primary data from Ghana and secondary data from Australia were used for the study. The Ghana Chamber of Mines is the primary minerals industry association in Ghana playing the advocacy and coordinating role in the minerals industry. Mining companies in Ghana were approached to participate through the Ghana Chamber of Mines. The Chamber facilitated access to the mining companies by endorsing and encouraging member organisations to participate in the study. Health and Safety Managers of each mine site were contacted to gain consent for participation. Mines that consented to participate were then contacted by one of the authors (WAD) who worked with the managers to determine logistical arrangements for data collection. Description of the Australian coal data can be found elsewhere [17].

### Patient and public involvement

No patients were involved in the study.

### Measures and scoring

The survey included information on socio-demographic characteristics.

#### Primary outcome variables

The Kessler Psychological Distress Scale (K10) was used to measure participants’ current level of mental health. It is one of the most widely used screening tools for detecting mental health problems [18]. Each item is scored from one ‘none of the time’ to five ‘all of the time’. Scores of the 10-items are then summed, yielding a minimum score of 10 and a maximum score of 50. Low scores indicate low levels of psychological distress and high scores indicate high levels of psychological distress. The Cronbach alpha of K10 was 0.86.

The Job Content Questionnaire (JCQ) was used to measure psychosocial job hazards affecting the psychological and physical well-being of mineworkers [19]. Scores of the 12 items ranged from 0 (not applicable) to 1 (often), higher score indicate more job dissatisfaction. The instrument has the following sub-scales; decision latitude (3 items), psychological demands (3 items), skills discretion (4 items) and social support (2 items). The Cronbach alpha was 0.64.

#### Secondary outcome variables

The Berkman Syme Social Network Index (SNI) measures the total number of people with whom the respondent has regular contact (i.e., at least once every 2 weeks) [20]. These include relationships with a spouse, parents, parents-in-law, children, other close family members, close neighbours, friends, workmates, schoolmates, fellow volunteers, members of groups without religious affiliation, and religious groups. One point is assigned for each type of relationship with possibility of 12 contact roles. The Cronbach alpha was 0.65.

The Alcohol Use Disorders Identification Test (AUDIT) is a 10-item screening tool developed by the World Health Organization (WHO) to assess alcohol consumption, drinking behaviours, and alcohol-related problems. Alcohol risk level was categorised into low-risk, risky or hazardous level, high-risk or harmful level and high-risk [21]. The Cronbach alpha was 0.82.

Help-seeking was measured by participants self-reporting the number of times they had consulted with various support people (e.g. psychiatrist, mental health nurse, general practitioner, drug and alcohol counsellor) to discuss their own mental health within the last 12 months [7].

Predictor variables: Country (Australia or Ghana).

Covariates for propensity score adjustment: Age ^1^, gender ^2^, education ^3^, shift type ^4^, years working at the mine ^5^, worried about losing job ^6^, audit stratum ^7^, relationship status ^8^, working in mining for financial reasons ^9^, social network index ^10^, travel time ^11^, work schedule ^12^, concern over losing job ^13^, employment category ^14^, financial reasons ^15^, SNI ^16^, K10 ^17^.

### Data collection procedures

The Australian data collection occurred during participants shift, or during pre-shift meetings. Participants were provided with a written information statement and a brief presentation outlining the purpose of the research. The detailed process of data collection in Australia is explained elsewhere [17]. A brief presentation was delivered to participants by WAD at each mine site in Ghana during the data collection period before information statement and surveys were distributed. The survey took approximately 20-30 min to complete with return of survey at designated collection places at each mine site. The participant information statement and consent forms stated that return of a survey implied consent. The period of the Ghana data collection was between December 2018 and March 2019 and the Australia data collection occurred between December 2013 and March 2015. Ethics approval for the study was provided by the University of Newcastle Human Ethics Committee (Approval Numbers H-2018-0194 and H-2013-0135) and ratified by the Ghana Chamber of Mines research committee (074/M2/18C).

### Statistical analysis

Statistical analysis was completed using STATA Version 17 [22] software and SAS software [23]. Country characteristics were summarised by using descriptive analysis and chi-square analysis. To identify the differences/similarities in psychological distress between mineworkers in Ghana and Australia, a crude logistic regression was performed, regressing psychological distress (moderate, high or very high scores) on country (Australia or Ghana), to estimate the odds of psychological distress in Ghana compared to Australia. Propensity scores were utilized to adjust these estimates, accounting for differences in the samples. Where able to, authors accounted for site as a random effect. In cases where model convergence could not be achieved with random effect, we report standard logistic regression results.

## Results

A total of 2622 participants; 1457 from Australia and 1165 from Ghana were sampled for this study as shown in Table 1. Chi-square tests were performed to determine associations between the variables.

**Table 1:** Sociodemographic data and outcome variables

Characteristic	Australia N (%)	Ghana N (%)	Chi square tests of independence
*Sociodemographic data*			
Country	1457 (56)	1165 (44)	
Gender			
Male	1266 (87)	1012 (87)	χ2 (1) = 0.23 *P* = 0.64 φ = 0.01 *n* = 2612
Female	181 (13)	153 (13)	
Age in years			
< 24	116 (8)	81 (7)	χ2 (4) = 91.82 *P* = 0.01 φ = 0.19 *n* = 2611
25 – 34	448 (31)	543 (47)	
35 – 44	446 (31)	344 (30)	
45 – 54	331 (23)	161 (14)	
55+	105 (7)	36 (3)	
Relationship status			
Single	133 (9)	299 (26)	χ2 (6) = 348.63 *P* = 0.01 φ = 0.37 *n* = 2610
Relationship (living apart)	72 (5)	122 (10)	
Separated	46 (3)	8 (1)	
Widowed	4 (0.3)	15 (1)	
Married	866 (60)	691 (59)	
Relationship (live together)	286 (20)	23 (2)	
Divorced	38 (3)	7 (1)	
Education			
No education	36 (2)	42 (4)	χ2 (5) = 588.08 *P* = 0.01 φ = 0.47 *n* = 2622
Year 10/ Junior High School	266 (18)	49 (4)	
Year 12/ Senior High School	175 (12)	143 (12)	
Trade/Apprenticeship	524 (36)	140 (12)	
Certificate/ Diploma	250 (17)	158 (14)	
University degree or higher	206 (14)	633 (54)	
Employment category			
Manager	68 (5)	80 (7)	χ2 (6) = 480.69 *P* = 0.01 φ = 0.43 *n* = 2621
Technicians/Trade/Labourer	562 (39)	344 (30)	
Professional	198 (14)	435 (37)	
Clerical	36 (2)	123 (11)	
Machinery operator	539 (37)	96 (8)	
Other	53 (4)	87 (7)	
Employment contract			
Permanent/ Ongoing basis	1231 (85)	314 (27)	χ2 (3) = 919.96 *P* = 0.01 φ = 0.59 *n* = 2616
Fixed term/ Casual	93 (6)	532 (46)	
Contractor/ Subcontractor	126 (9)	299 (26)	
Other	1 (0.1)	20 (2)	
Work Schedule			
Regular Shift	697 (48)	825 (71)	χ2 (1) = 136.55 *P* = 0.01 φ = −0.23 *n* = 2613
Rotating Shift	751 (52)	340 (29)	
*Primary outcome variables*			
JCQ			
Job control equal or outweigh job demand	766 (57)	581 (43)	χ2 (1) = 4.64 *P* = 0.03 φ = 0.04 *n* = 2470
Job demands outweigh Job control	590 (53)	533 (47)	
K10			
Low or moderate	1264 (87)	1012 (87)	χ2 (1) = 0.10 *P* = 0.75 φ = 0.00 *n* = 2613
High or very high	184 (13)	153 (13)	
*Secondary outcome variables*			
AUDIT Stratum			
No known risk	820 (58)	1077 (93)	χ2 (3) = 405.56 *P* = 0.01 φ = 0.40 *n* = 2567
Risky or hazardous	454 (32)	66 (6)	
High risk or harmful	76 (5)	8 (1)	
High risk, dependence likely	61 (4)	5 (0.4)	
SNI	375 (82)	81 (17.76)	χ2 (3) = 329.70 *P* = 0.01 φ = 0.38 *n* = 2336
	581 (52.39)	528 (47.61)	
	229 (65.24)	122 (34.76)	
	96 (22.86)	324 (77.14)	
Help-Seeking Behaviour			
General Practitioner			
0 times	1163 (81)	979 (84)	χ2 (1) = 5.04 *P* = 0.03 *n* = 2600
1 + times	275 (19)	183 (16)	
Psychiatrist			
0 times	1392 (97)	1133 (98)	χ2 (1) = 0.33 *P* = 0.57 *n* = 2595
1 + times	41 (3)	29 (2)	
Psychologist			
0 times	1339 (93)	1122 (97)	χ2 (1) = 12.73 *P* = 0.01 n = 2595
1 + times	94 (7)	40 (3)	
*Clergy			
0 times	1416 (99)	923 (79)	χ2 (1) = 268.40 *P* = 0.01 *n* = 2596
1 + times	18 (1)	239 (21)	
Friends or Family			
0 times	855 (60)	626 (54)	χ2 (1) = 8.66 *P* = 0.01 *n* = 2596
1 + times	579 (40)	536 (46)	
Chemist			
0 times	1370 (96)	1039 (89)	χ2 (1) = 35.24 *P* = 0.01 *n* = 2595
1 + times	64 (4)	122 (11)	

Notes: χ2 = chi-square statistic; *p* < 0.05; φ = phi; n = sample size *also referred to as pastoral care, faith healers & prophet

A higher proportion of Australians reported job control equal or outweighing job demands than Ghanaians (57% compared to 53% respectively). JCQ was significant Chi^2^ (1) = 4.64, *N* = 2470, *p* = 0.03 but there was no significant difference between country and K10, Chi2 (1) = 0.10, *N* = 2613, *p* = 0.75. Country and AUDIT was significant, Chi-square (3) = 405.56, *p* = 0.01. Australians reported higher proportions of risky or hazardous alcohol use, and high risk or harmful and high-risk dependence than Ghanaians (41% compared to 7.4%, *p* = 0.1). Differences in SNI were also statistically significant (chi-square (3) = 329.70, *p* = 0.01). Regarding help-seeking, there was a significant difference between country and help sought. Whereas a higher proportion of Australians reported consulting a GP and psychologist in the past 12 months about mental health problems, Ghanaians consulted the clergy, family or friends and chemist.

### Propensity score analysis

The propensity score analyses were utilised to estimate the differences between Ghana and Australia in terms of their association with each outcome, after adjusting for differences in the characteristics of the populations that may also be associated with the outcome. The association between K10 (high/very high vs low/medium) and country was investigated using a) an adjusted logistic regression model, b) a stabilised propensity score logistic regression model. The stabilised version was presented due to an imbalance in the propensity scores, largely due to the inclusion of the AUDIT as a covariate (with only 6.8% of Ghanaians reporting risky levels of drinking, compared to 41.8% of Australians).

Psychological distress (K10): Covariates ^7, 8, 13, 16^ were included in the final propensity score. The results of the unadjusted and stabilized propensity score adjusted logistic regressions are reported in Table 2. Unadjusted for confounders, there was no difference in the odds of reporting high psychological distress between Ghana and Australia (OR = 1.04, *p* = 0.75). After adjusting for potential confounders, Ghanaians had increased psychological distress than Australians (OR = 7.75, *p* = 0.01).

**Table 2:** Propensity score analysis for outcome variables

		OR (95% CI)	*P*-value
K10 (Odds that k10 = high or very high)			
Unadjusted	Ghana	1.04(0.83, 1.31)	0.75
	Australia	ref	–
Stabilised Propensity score adjusted	Ghana	7.75(6.43, 9.34)	0.01
	Australia	ref	–
AUDIT: (odds that Audit risk = risky drinking (Audit stratum = 1))			
Unadjusted	Ghana	0.10(0.08, 0.13)	< 0.01
	Australia	ref	–
JCQ (Odds of JCQ ratio = job demands outweigh control)			
Unadjusted	Ghana	*1.17(0.94, 1.45)	*0.17
	Australia	Ref	–
Stabilised Propensity score adjusted	Ghana	*1.54(1.02, 2.32)	* < 0.04
	Australia	ref	–
SNI (modelling the odds of scoring medium, medium high or high vs low)			
Unadjusted	Ghana	*4.98(3.80, 6.55)	* < 0.01
	Australia	ref	–
Stabilised Propensity score adjusted	Ghana	*5.21(1.97, 13.77)	* < 0.01
	Australia	ref	–

*models where random effects could be used

#### Audit

Covariates ^9, 10, 11, 12, 13, 14^ were included in the final propensity score. The results of the unadjusted and stabilized propensity score adjusted logistic regressions are reported in Table 2. A propensity score analysis was not able to be performed for the AUDIT outcome, as far too few Ghanaians drank at risk levels to ensure the results would be stable, and therefore balance could not be achieved. Unadjusted, Ghanaians were significantly less likely to drink at risky levels than Australians (OR = 0.10, *p* < 0.01).

#### JCQ

Covariates^3, 4, 7, 12, 13, 14, 17^ were included in the final propensity score. The results of the unadjusted and stabilized propensity score adjusted logistic regressions are reported in Table 2. Prior to adjustment, Ghanaians were more likely to report their job demands outweighed job control (OR = 0.19, *p* = 0.03). After propensity score adjustment, this increased (OR = 1.62, *p* < 0.01).

#### SNI

Covariates ^1, 7, 8, 12, 13, 17^ were included in the final propensity score. The results of the unadjusted and stabilized propensity score adjusted logistic regressions are reported in Table 2. Prior to adjustment, Ghanaians were more likely to score medium/high/very high on the SNI than Australians (OR = 4.98, *p* < 0.01), but after adjustment this reduced to OR = 1.98 (*p* < 0.01).

Significant interaction terms were included in a multivariable model to determine predictors of psychological distress across country.

From the multivariable model in Table 3, for both countries, JCQ was not associated with psychological distress. Alcohol predicted higher psychological distress among Ghanaian mineworkers who drank at risky or hazardous alcohol levels (OR = 2.13, *p* = 0.04). Mineworkers in both countries who were worried about losing their jobs reported increased psychological distress. Similarly, those who were working in mining solely for financial reasons also reported increased psychological distress. Mineworkers across both countries working 8 h reported low psychological distress. There is almost a dose response effect, with lowest odds for 8 h (OR = 0.45), then 9-11 h (OR = 0.69), then 12 h (OR = 0.78) shift length. Except that those who worked < 8 h had almost twice the odds than > 12 h.

**Table 3:** Predictors of Psychological Distress across Country

K10			Odds Ratio (95% CI)	*P*-value
**Source**	Ghana		1.05 (0.54, 2.06)	0.88
	Australia		(ref)	.
**JCQ Ratio**	Job demands outweigh Job control		1.05 (0.79, 1.39)	0.73
	Job control equal or outweigh job demands		(ref)	.
**SNI (dich.)**	Higher SNI score (> 0)		0.96 (0.66, 1.39)	0.82
	Low SNI score (0)		(ref)	.
**AUDIT risk**	Drinking at risky levels		1.58 (1.11, 2.27)	0.01
	Non risky drinking		(ref)	.
Source*AUDIT risk	Ghana	Drinking at risky levels	2.13 (1.04, 4.38)	0.04
	Ghana	Non risky drinking	(ref)	.
	Australia	Drinking at risky levels	(ref)	.
	Australia	Non risky drinking	(ref)	.
**Worried about losing job**	Moderately-extremely worried		1.71 (1.30, 2.27)	0.01
	Not at all/mildly worried		(ref)	.
**Relationship Status**	Divorced		0.68 (0.25, 1.88)	0.46
	In a relationship (Not living together)		1.09 (0.39, 3.05)	0.87
	In a relationship (living together)		1.18 (0.39, 3.54)	0.77
	Married		0.49 (0.18, 1.34)	0.16
	Separated (but not divorced)		0.52 (0.20, 1.33)	0.17
	Single (Never Married)		0.73 (0.27, 1.98)	0.54
	Widowed		(ref)	.
**Financial Reasons**			1.47 (1.25, 1.72)	<.01
**Age**			1.15 (0.98, 1.36)	0.09
**Gender**	Female		1.37 (0.89, 2.10)	0.15
	Male		(ref)	.
**Common shift**	Less than 8 h		2.08 (0.35, 12.20)	0.41
	8 h		0.45 (0.26, 0.77)	0.01
	9-11 h		0.69 (0.45, 1.05)	0.08
	12 h		0.78 (0.52, 1.16)	0.22
	More than 12 h		(ref)	–
**Work Schedule**	Regular		1.16 (0.83, 1.61)	0.39
	Rotating		(ref)	.
**Education**	Certificate/diploma		0.85 (0.50, 1.44)	0.54
	No education		0.26 (0.07, 0.96)	0.04
	Trade/apprenticeship		0.65 (0.40, 1.06)	0.09
	University degree or higher		0.89 (0.52, 1.50)	0.65
	Year 10/Junior Secondary		0.67 (0.37, 1.20)	0.18
	Year 12/Senior Secondary		(ref)	.
**Employment category**	Other		0.97 (0.66, 1.43)	0.89
	Professional/clerical		(ref)	.
**Employment contract**	Contractor		0.88 (0.57, 1.36)	0.58
	Fixed term/casual/other		0.51 (0.17, 1.53)	0.23
	Permanent		(ref)	.

The results of the logistic regression looking at the differential effect of JCQ, SNI and AUDIT across country are reported in Table 4.

**Table 4:** Differential effect of JCQ, SNI, AUDIT across country

*Odds of risky drinking for the differential effect of JCQ across country*				
Country	**JCQ level**	**Reference group**	**Odds Ratio (95% CI)**	***p*-value**
Ghana	Job demands > Job control	Job control ≥ job demands	*0.88 (0.55, 1.41)	*0.60
Australia	Job demands > Job control	Job control ≥ job demands	*1.26 (1.01, 1.57)	*0.04
*Odds of psychological distress for the differential effect of Social Network Index across country*				
Country	**SNI**	**Reference group**	**Odds Ratio (95% CI)**	***p*-value**
Ghana	Higher SNI score (> 0)	Low SNI score (0)	0.54 (0.31, 0.96)	0.04
Australia	Higher SNI score (> 0)	Low SNI score (0)	0.67 (0.48, 0.93)	0.02
*Odds of risky drinking for the differential effect of Social Network Index across country*				
Country	**SNI**	**Reference group**	**Odds Ratio (95% CI)**	***P*-value**
Ghana	Higher SNI score (> 0)	Low SNI score (0)	*0.44 (0.22, 0.86)	*0.02
Australia	Higher SNI score (> 0)	Low SNI score (0)	*0.98 (0.76, 1.25)	*0.84

*models where random effects could be used

The results of the logistic regression looking at the differential effect of the JCQ and SNI across country is reported in Table 4. Among Ghanaians, job demands outweighing control was associated with risky or hazardous alcohol levels (although not significant) and among Australians it was associated with increased odds of risky or hazardous alcohol levels (1.26, *p* = 0.04). Among Ghanaians, higher SNI was associated with decreased odds of risky or hazardous alcohol levels compared to a low SNI. Among both Ghanaians and Australians, higher SNI was associated with lower levels of psychological distress.

## Discussion

This study is the first to compare the patterns of psychological distress, job demand-control, and associated characteristics among mineworkers in Ghana and Australia. Ghanaian mineworkers were slightly younger, aged 25-34 years although both countries had majority of the mineworkers aged 25-44 years. A difference was observed in the educational qualification and employment category of both countries; Ghanaians had more tertiary education qualifications compared to their Australian counterparts’ majority of who had trade/apprenticeship qualifications. This difference reflects the training in both countries. The University of Mines and Technology (UMaT) in Ghana is a tertiary institution in Ghana located in the Tarkwa region (the hub of mining in Ghana) and offering courses specific to the extractive industry (i.e., mining, oil and gas etc.), thus accounting for a large number of mineworkers in Ghana having tertiary education qualifications [24]. In contrast, Australians have vocational training from the Technical and Further Education college (TAFE) [25]. In terms of employment category, Ghanaian mining companies are moving from owner mining to contract mining to reduce costs, hence more contract staff being employed [6]. It is therefore not surprising that compared to Australia, majority of the Ghanaians were on fixed-term contract or contractors/sub-contractors. The mining industry like most extractive industries is an entrapped sector with the technical skills not easily transferable, thus, mine workers have little option in the current dispensation of contract mining to go for contracts and still stay relevant.

Ghanaian mineworkers were more likely to report job demands (e.g., long working hours, time pressure, huge workload) outweighing control compared to Australian mineworkers. This finding is consistent with previous mining studies in Ghana where psychological job demands were higher among mineworkers [26]. Long working hours in general have been associated with increased risk of mental health problems across different occupations [27]. The mining work is associated with long shift patterns, high job demands, physical factors such as heat and noise hazards which all affect the mental health of workers [26, 27]. Despite job demands outweighing control, this was associated with lower risk of psychological distress in the Ghanaian mineworkers but among the Australians, it was associated with higher risk of psychological distress. The finding is consistent with a study in Ghana and the UK; despite increased job demands, Ghanaian workers viewed it less negatively than UK workers and are able to manage the job demands of the job [28]. With a relatively high unemployment rates in Ghana, workers may likely endure adverse working conditions such as increased job demands [29]. Even with increased job demands, the mineworkers petitioned the Minerals Commission of Ghana (regulator of minerals control in Ghana) to maintain overtime working hours at the mine instead of working 8 h a day or 40 h in 7 days [30]. An explanation for Ghanaian mineworkers working overtime may be to gain extra income and maintain the mineworker lifestyle even as contract workers. This lifestyle has been termed the ‘golden handcuffs’ referring to high remuneration in the Australian mining industry resulting in mineworkers becoming dependent on the salary [17]. Similar findings have been reported among mineworkers working overtime to supplement their income which contributes to fatigue [31].

Also, the uncertainty surrounding contract work is a stimulator to work extra hours to make extra income for future eventualities. Increased job demands may not be interpreted as having adverse job outcomes in Ghana. Considering that most of the mining companies in Ghana are moving towards contract staffing and several participants in the study fall within this category of employment, this finding may not be surprising as contract staff have little bargaining power as they fear losing their contract if they complain about the job demands. Thus, enduring rather than complaining. In contrast, in Australia, workers may have stronger bargaining capacity to negotiate work schedules and more control over their working time [32]. Alternatively, Australian mineworkers may have higher income which suggests less need to take on heavy workload and be exempt from overtime [33]. Job control therefore may not be a universal factor but dependent on cultural settings. Expectations of job control are higher in Australia especially because there is greater policy and legislation around the workplace, but Ghanaian mineworkers may have lower expectations of the work environment. Nevertheless, an imbalance between job demands and control in any work could exhaust workers, leading to poorer mental health outcomes and unsafe work behaviour as well as injuries even if workers do not acknowledge it [34].

In both countries, mineworkers who worried about losing jobs and working solely because of financial reasons had high psychological distress. Working solely because of remuneration or financial expectations does not lead to fulfilment or satisfaction but rather to psychological distress when this goal is not attained. Mineworkers in both countries working 8 h reported low psychological distress but those who worked less than 8 h had a higher risk of psychological distress. This maybe reflective of underemployment hence less remuneration [35].

Drinking pattern was one of the main indicators of the differences between the countries. Australian mineworkers drank alcohol at risky or hazardous levels than Ghanaian mineworkers [36, 37]. The reflection of the risky or hazardous alcohol levels at the workplace may reflect a drinking culture prevalent in Australia [38]. The very low risky or hazardous alcohol levels among Ghanaian mineworkers may be due to cultural, religious and educational reasons -majority were religious and highly educated [39]. Interestingly, even though Ghanaians were less likely to drink at risky or hazardous alcohol levels (see Table 2), for those that did, the risk of psychological distress was higher than for Australians who drank at risky or hazardous alcohol levels. Although Ghanaian mineworkers who had job demands outweighing job control did not report increased psychological distress, increased risky or hazardous alcohol levels was found. In Australia increased risky or hazardous alcohol levels was also found among mineworkers with high job demand and low job control. Both countries used alcohol as a form of coping mechanism with increased job demands, but in Ghana this association was not significant. It is possible that this association may be real among the Ghanaian mineworkers but not significant because of the low report of risky levels alcohol. As stated earlier, low psychological distress was reported among Ghanaian mineworkers although job demands outweighed job resources. It is therefore possible that the use of alcohol to cope with job demands may be a response to the physical qualities (which was not measured) of the work environment and not necessarily the psychological demands of the work. Irrespective of this, excessive risky or hazardous alcohol levels is an extreme behaviour in the Ghanaian setting therefore this may be an important signal of underlying emotional or psychological problems for mineworkers [40].

Furthermore, Ghanaian mineworkers reported high social network and frequent contacts than Australians. For both countries, higher social network was associated with lower risk of psychological distress and in Ghana, high social network was associated with lower risky alcohol levels. Informal relationships are part of the traditional Ghanaian family and culture providing emotional and moral support [41]. Compared to Australian mineworkers, Ghanaian mineworkers’ preference of help for mental health problems was mostly non-professional help such as the clergy, family/friends, and the chemist. Therefore, Ghanaian mineworkers who had such informal support to cope lowered their risk of risky or hazardous alcohol levels. Australians sought help from both professional and non-professional sources such as the GP, psychiatrist, psychologist, and family/friends. These findings observed reflect cultural differences between countries. Generally, in Ghana, mental health services are limited hence a significant number of people may prefer alternative pathways to care such as the clergy who are easily accessible and affordable [42]. Consistent with previous studies in Australia, the GP was the most common source of help contacted among Australian mineworkers [43]. Both the GP and clergy may serve as gatekeepers for mental health in both Australia and Ghana respectively, especially when faith-based organisations can be beneficial in responding to this shortage of mental health workers in Ghana [44].

### Theoretical and practical implications

The Job Demand-Control (JDC) model reasons that high job control will ameliorate the experience of psychological distress, but from the findings of this study, in the Ghanaian population, although job demand outweighed job resources, it was not associated with psychological distress. This could be attributed to the changing phase of mining in Ghana, which utilise contract staff as opposed to permanent staff. This situation has the potential to influence these contract staff not to exhibit any sign of psychological distress even under extreme work demands for fear of not having their contracts renewed if they did so. Also, with the qualifications of the Ghanaian mineworkers, it is likely they have personal resources to deal with excessive work demands even in the absence of organisational resources as explained by the Job Demand-Resource model. Furthermore, the job control dimensions need to be evaluated independently to further understand its relation to psychological distress. A re-examination of job demand and job control in the mining industry prior to making any changes is recommended. Specific risk factors such as shift schedules, rosters, and job roles need to be considered in relation to their impact on employee mental health.

The findings on the social network of mineworkers should inform the selection of services and programs suitable to this industry. In Australia, peer-based programs have been effective in promoting early help-seeking [45, 46]. A similar multicomponent program is recommended in Ghana which would be integrated with chaplaincy services and faith-based programs. The chaplain could then become a gatekeeper and a referral pathway to mental health professionals promoting improved physical and psychological work environments.

An investment into mental health research across the resource industry will help to build evidence, develop policies, and design tailored interventions. Collaboration and strategic partnerships with academic stakeholders to undertake further research in mental health in the resources sector is needed. Industry and academia collaboration is key to building new knowledge through research and developing innovative solutions.

### Recommendations

It is important that work structures, design, and policies in Ghanaian mining companies consider physical, psychological, social, organisational, and cultural factors that promote mental well-being of mineworkers. Both countries may benefit from implementing health promotion programmes as part of health and safety policies which include a focus on alcohol use outside of the workplace. In Ghana, both professionals and non-professionals can act as mental health gatekeepers as faith-based organisations can be beneficial in responding to the shortage of mental health workers.

In terms of policy development, the study highlights the importance of an integrated occupational health and safety policy, which considers mental health related issues and other psychosocial issues at the workplace as integral parts of the quest to promote the highest form of physical, mental and social well-being of mineworkers. This should be considered at the enterprise as well as the national levels in Ghana to give wholeness to the practice of sound occupational health and safety, which is touted in the mining industry.

The study provides empirical evidence in supporting learnings across countries; the differences between countries are indicative of the need to consider contextual factors in both countries since both hosts multinational mining companies. For instance, the concept such as flexible work arrangements are foreign to Ghana and caution must be taken when transfer of human resource from a western country is involved. Currently the mining industry in Australia has developed and incorporated mental health programs and interventions in the health and safety policy, which provides an opportunity to guide similar in the Ghanaian context. Further a multicomponent framework that promotes peer and manager support as part of the broader organisational support should be encouraged. The data also supports the significance of the role of professionals and non-professionals in the delivery or mental health care providing a referral pathway for further assessment. Further studies in identifying specific workplace factors leading to increased job demands are required.

### Limitations

It is acknowledged the Australian data was collected 6 years prior to the current study. Factors which impacted mental health, job demand and job control may have changed in this period. Due to the less risky or hazardous alcohol levels among Ghanaian mineworkers, AUDIT might have skewed the data, but it was important to include in the models.

## Conclusion

In conclusion, the study findings advance our understanding of peculiar cultural factors present in each country and help identify important differences in Australia and Ghana. This study builds evidence on the need for the development of appropriate context specific interventions for mental health through industry/organisational commitment and policy support, leading to health and economic benefits.

## Data Availability

The datasets used and/or analysed during the current study are available from the corresponding author on reasonable request. The dataset is part of the first author’s PhD thesis and has not been published publicly yet by the University.
